# rs929387 of *GLI3* Is Involved in Tooth Agenesis in Chinese Han Population

**DOI:** 10.1371/journal.pone.0080860

**Published:** 2013-11-20

**Authors:** Haochen Liu, Dong Han, Singwai Wong, Xu Nan, Hongshan Zhao, Hailan Feng

**Affiliations:** 1 Department of Prosthodontics, Peking University School and Hospital of Stomatology, Beijing, China; 2 Department of Medical Genetics, Peking University Health Science Center, Beijing, China; 3 Peking University Center for Human Disease Genomics, Peking University Health Science Center, Beijing, China; East Carolina University, United States of America

## Abstract

Tooth agenesis is one of the most common anomalies of human dentition. Recent studies suggest that a number of genes are related to both syndromic and non-syndromic forms of hypodontia. In a previous study, we observed that polymorphism in rs929387 of *GLI3* might be associated with hypodontia in the Chinese Han population based on a limited population. To further confirm this observation, in this study, we employed 89 individuals diagnosed with sporadic non-syndromic oligodontia (40 males and 49 females) to investigate the relationship between polymorphism in rs929387 of *GLI3* and tooth agenesis. These individuals were analyzed with 273 subjects (125 males and 148 females) diagnosed with non-syndromic hypodontia and 200 healthy control subjects (100 males and 100 females). DNA was obtained from whole blood or saliva samples and genotyping was performed by a Matrix-Assisted Laser Desorption/Ionization Time of Flight Mass Spectrometry (MALDI-TOF MS) method. Significant differences were observed in the allele and genotype frequencies of rs929387 of *GLI3*. Distributions of genotypes TT, TC and CC of rs929387 polymorphism were significantly different between the case group and the control group (*P* = 0.013) and C allelic frequency was higher in case group [*P* = 0.002, OR = 1.690, 95% CI (1.200-2.379)]. Additionally, our analysis shows that this difference is more pronounced when compared between the male case group and the male control group. The function study suggests that variation in GLI3 caused by rs929387 leads to a decrease in its transcriptional activity. These data demonstrated an association between rs929387 of *GLI3* and non-syndromic tooth agenesis in Chinese Han individuals. This information may provide further understanding of the molecular mechanisms of tooth agenesis. Furthermore, *GLI3* can be regarded as a marker gene for the risk of tooth agenesis.

## Introduction

Permanent tooth agenesis is one of the most common dental developmental anomalies in human [1]. The prevalence of dental agenesis of permanent teeth ranges from 2.2 to 10.1% in the general population excluding third molars [2]. The majority of persons are missing only one or two teeth and hypodontia is often used as a collective term to describe the absence of one to six teeth excluding third molars [3,4]. Oligodontia refers to the absence of more than six teeth, excluding third molars [5]. Tooth agenesis may present as part of a syndrome, however, the non-syndromic form is more common.

Several studies suggested that tooth agenesis is mainly caused by genetic factors [1,3,6–9]. To date, mutations in *AXIN2* (axis inhibition protein 2), *EDA* (ectodysplasin A), *MSX1* (msh homeobox 1), *PAX9* (paired box 9) and *WNT10A* (wingless-type MMTV integration site family, member 10a) have been demonstrated to be associated with non-syndromic tooth agenesis [10–16]. However, there are still many cases that could not be identified mutations in these five genes. Furthermore, the etiological mechanism of non-syndromic tooth agenesis remains unclear.

Tooth development is a very complicated process involving many genes and signaling pathways [17]. Certain alterations in one or more of these genes may cause tooth agenesis [1]. Several studies suggest that gene polymorphisms may cause disease susceptibility [18,19]. Single nucleotide changes, which occur at a high frequency in the human genome, are the most common polymorphisms and may affect the function of genes. Thus, single nucleotide polymorphisms (SNPs) may be a risk factor for non-syndormic tooth agenesis.

In a previous study, we observed that polymorphism in rs929387 of *GLI3* might be associated with hypodontia in Han population [20]. However, that is only based on a limited population. In this study, we collected individuals diagnosed with non-syndromic oligodontia and studied the two type of population (individuals with non-syndromic hypodontia and individuals with non-syndromic oligodontia) together. Our results show that polymorphism in rs929387 of *GLI3* associated with tooth agenesis in Chinese Han population, especially in male. We further employed functional study to test whether variation caused by rs929387 could affect the function of GLI3. 

## Materials and Methods

### Subject selection and sampling

This study was approved by the Institutional Review Board of Peking University School and Hospital of Stomatology. A total of 562 individual subjects were analyzed in this study, which include 89 subjects (40 males and 49 females) diagnosed with sporadic non-syndromic oligodontia, 273 subjects (125 males and 148 females) diagnosed with sporadic non-syndromic hypodontia (excluding the third molar) and 200 healthy control subjects (100 males and 100 females). All individuals participating were genetically unrelated ethnic Han Chinese from Beijing or the surrounding regions. 

All subjects didn’t have a history of teeth extraction or loss. Naturally missing teeth within the adult dentition were confirmed by X-ray examination and no other dental anomalies were observed in any subjects. All of participants provided their written informed consent to participate in this study. Blood samples and oral swabs were coded to maintain confidentiality. Genomic DNA of participants with tooth agenesis were extracted from peripheral blood lymphocytes using the the TIANamp Blood DNA kit (Tiangen, Beijing, China) according to manufacturers’ instruction. DNA samples of the normal volunteers were extracted from buccal epithelial cells using the TIANamp Swab DNA kit (Tiangen, Beijing, China) according to the manufacturers’ instructions.

### Polymorphism genotyping

Primers for polymerase chain reactions (PCR) and single base extensions were designed using the Assay Designer software package (Sequenom, Inc, San Diego, CA). The forward primer was 5’-ACGTTGGATGTCGCTGGCCCTCCTCAC-3’ and the reverse primer was 5’-ACGTTGGATGATGCCCCGAGGAGGTG-3’. SNP genotyping was performed using the MassARRAY system (Sequenom) by a Matrix-Assisted Laser Desorption/Ionization Time of Flight Mass Spectrometry (MALDI-TOF MS) method according to manufacturer’s instruction. Completed genotyping reactions were spotted onto a 384-well spectroCHIP (Sequenom) using the MassARRAY system (Sequenom) and determined by MALDI-TOF-MS. Genotype calling was performed in real time using MassARRAY RT software version 3.0.0.4 and analyzed using the MassARRAY Typer software version 3.4 (Sequenom).

### DNA constructs

The expression vector pCMV6-GLI3 with the c-Myc epitope tags was purchased from OriGene Technologies, Inc. *In vitro* site-directed mutagenesis was performed to construct pCMV6-P998LGLI3 by using the QuikChange Lightning Site-Directed Mutagenesis Kit (Stratagene Corp., La Jolla, CA, USA). The mutated constructs were verified by sequencing the whole vectors. Eight directly repeated copies of a GLI-binding site (GLI-BS: 5’-GAACACCCA-3’) fragment were subcloned into the pGL3-Basic vector (Promega) upstream of the firefly luciferase reporter gene [21]. 

### Cell culture, transient transfection and luciferase reporter assay

HeLa cells were seeded with 1×10^5^ per well in a 6-well plate for 24 hours prior to transfection. After overnight incubation, cells were transfected with Lipofectamine 2000 (Invitrogen) according to the manufacturer’s instruction. GLI3 expression vectors (pCMV6-GLI3 or pCMV6-P998LGLI3) were co-transfected with pGL3-GLI-BS Luc reporter plasmid; the phRL-TK plasmid (Promega) was used as the internal control. Cell extracts were prepared using the Cell Culture Lysis Reagent (Promega) 48 h after transfection and the extracts were assayed by using Dual-Luciferase Reporter Assay System (Promega). Firefly luciferase activity was normalized based on Renilla luciferase activity. All reporter assays were repeated at least three times. Data shown are average values ±SD from one representative experiment.

### Protein preparation and Western blot analysis

Cells were washed twice with ice-cold phosphate-buffered saline (PBS) and lysed in RIPA lysis buffer (50 mM Tris–HCl, pH 7.4; 150 mM NaCl; 1% deoxycholate Na; 1% NP-40; 0.1% sodium dodecylsulfate, with freshly added protease inhibitor cocktail) for 30 min at 4 °C. Cell lysates were clarified by centrifugation at 4 °C at 16000g for 20 min. Protein concentrations were determined using the BCA protein assay reagent (Pierce, USA). Equal amounts of protein were electrophoresed by SDS–PAGE and transferred onto a nitrocellulose membrane (Amersham Pharmacia, UK). Membranes were blocked in Tris-buffered saline containing 0.1% Tween-20 (TBST) and 5% nonfat milk and then incubated overnight at 4 °C with the appropriate primary antibody. After washing in TBST buffer, the membranes were incubated for 1 h with the corresponding IRDye™ 700-conjugated secondary antibody. The blots were scanned using an Odyssey Imaging System (LI-COR Bioscience). Primary antibodies were purchased from the following commercial sources: Mouse monoclonal antibodies against Myc (Sigma–Aldrich, St. Louis, MO); polyclonal antibody against β-actin. (Cell Signaling, Beverly, MA, USA).

### Statistical analysis

Chi square was used to test if genotype distributions were in Hardy-Weinberg equilibrium. Clinical information and gender was compared across genotypes, using chi-square tests. When *p*-values is lower than 0.05, it was considered statistically significant. The associations between genotypes and the risk of tooth agenesis were estimated by computing the odds ratio (OR) and their 95% confidence intervals (95%CI) from logistic regression analyses. The results of luciferase reporter assay were expressed as mean±SD of triplicate independent experiments. The data were analyzed by Student’s t-test. All statistical tests for this analysis were performed using SPSS 13.0 software.

## Results

### Polymorphism in rs929387 is associated with tooth agenesis in Han population

According to the number of missing teeth, general tooth agenesis was divided into two groups: 1) hypodontia group refers absence of one to six teeth and 2) oligodontia group refers absence of more than six teeth. Table 1 clearly shows that the distribution of genotype and allele was significantly different among different groups. The CC and TT genotype frequencies were 6.7% and 61.9% in the hypodontia group, 5.6% and 55.1% in the oligodontia group, and 2.9% and 72.6% in the control group, respectively. The distribution of genotype exhibited significant differences in the tooth agenesis cases (hypodontia vs. control, *P*=0.039; oligodontia vs. control, *P*=0.016; hypodontia+oligodontia vs. control, *P*=0.016). Compared with control group, combined CC and TC genotypes also showed significant differences in hypodontia group (*P*=0.022), oligodontia group (*P*=0.004) and the general tooth agenesis case group (*P*=0.005). Compared with an allele frequency of 15.1% in control group, the frequency of allele “C” was 22.4% in hypodontia group and 25.3% in oligodontia group (hypodontia vs. control, *P*=0.0.008; oligodontia vs. control, *P*=0.005; hypodontia+oligodontia vs. control, *P*=0.002). 

**Table 1:** Genotype and allele frequencies of the rs929387 in hypodontia, oligodontia and normal individuals.

	Hypodontia	Oligodontia	Control	*P*-value^a^	OR(95%CI)a^a^	*P* -value^b^	OR(95%CI)^b^	*P*-value^c^	OR(95%CI)^c^
Genotype									
TT	156(61.9%)	49(55.1%)	127(72.6%)						
TC	79(31.4%)	35(39.3%)	43(24.5%)	**0.039**		**0.016**		**0.013**	
CC	17(6.7%)	5(5.6%)	5(2.9%)						
CT+CC	96(38.1%)	40(44.9%)	48(27.4%)	**0.022**	1.628(1.072-2.474)	**0.004**	2.160(1.267-3.683)	**0.005**	1.755(1.181-2.610)
Allele									
T	391(77.6%)	133(74.7%)	297(84.9%)						
C	113(22.4%)	45(25.3%)	53(15.1%)	**0.008**	1.620(1.130-2.320)	**0.005**	1.896(1.213-2.964)	**0.002**	1.690(1.200-2.379)

^a^Hypodontia vs. Control; ^b^bOligodontia vs. Control; ^c^Hypodontia+Oligodontia vs. Control; OR: Odd Ratio; CI: confidence intervals; *P*-values lower than 0.05 were written bold.

Then we investigated the distribution of genotype and allele in different gender groups. For male, both alleles and genotype frequencies exhibited significant differences among different groups (Table 2). CC and TC showed higher frequency in male hypodontia group and male oligodontia group than male control group (hypodontia vs. control, *P*=0.033; oligodontia vs. control, *P*=0.0.004; hypodontia+oligodontia vs. control, *P*=0.007). Compared with male control group, combined CC and TC genotypes also showed significant differences in male hypodontia group (*P*=0.020), male oligodontia group (*P*=0.001) and the male case group(*P*=0.003). Additionally, compared with an allele frequency of 14.9% in male control group, the frequency of allele “C” was 26.1% in male hypodontia group and 25.3% male oligodontia group(hypodontia vs. control, *P*=0.007; oligodontia vs. control, *P*=0.001; hypodontia+oligodontia vs. control, *P*=0.001). However, no significantly statistical differences were observed among female groups (Table 3).

**Table 2:** Genotype and allele frequencies of the rs929387 in hypodontia, oligodontia and normal male individuals.

	Hypodontia	Oligodontia	Control	*P*-value^a^	OR(95%CI)a^a^	*P* -value^b^	OR(95%CI) ^b^	*P* -value^c^	OR(95%CI) ^c^
Genotype									
TT	65(56.5%)	17(42.5%)	63(72.4%)						
TC	40(34.8%)	20(50.0%)	22(25.3%)	**0.033**		**0.004**		**0.007**	
CC	10(8.7%)	3(7.5%)	2(2.3%)						
CT+CC	50(43.5%)	23(57.5%)	24(27.6%)	**0.020**	2.091(1.111-3.670)	**0.001**	3.551(1.622-7.775)	**0.003**	2.337(1.327-4.116)
Allele									
T	170(73.9%)	54(67.5%)	148(85.1%)						
C	60(26.1%)	26(32.5%)	26(14.9%)	**0.007**	2.009(1.206-3.346)	**0.001**	2.741(1.465-5.128)	**0.001**	2.185(1.345-3.551)

^a^Hypodontia vs. Control; ^b^Oligodontia vs. Control; ^c^Hypodontia+Oligodontia vs. Control; OR: Odd Ratio; CI: confidence intervals; *P*-values lower than 0.05 were written bold.

**Table 3:** Genotype and allele frequencies of the rs929387 in hypodontia, oligodontia and normal female individuals.

	Hypodontia	Oligodontia	Control	*P*-value^a^	OR(95%CI)^a^	*P*-value^b^	OR(95%CI) ^b^	*P*-value^c^	OR(95%CI) ^c^
Genotype									
TT	91(66.4%)	32(65.3%)	64(72.7%)						
TC	39(28.5%)	15(30.6%)	21(23.9%)	0.582		0.660		0.537	
CC	7(5.1%)	2(4.1%)	3(3.4%)						
CT+CC	46(33.6%)	17(34.7%)	24(27.3%)	0.319	1.348(0.749-2.427)	0.363	1.417(0.668-3.006)	0.273	1.366(0.781-2.388)
Allele									
T	221(80.7%)	79(80.6%)	149(84.7%)						
C	53(19.3%)	19(19.4%)	27(15.3%)	0.279	1.323(0.796-2.199)	0.390	1.327(0.695-2.535)	0.254	1.324(0.816-2.149)

^a^Hypodontia vs. Control; ^b^Oligodontia vs. Control; ^c^Hypodontia+Oligodontia vs. Control; OR: Odd Ratio; CI: confidence intervals; *P*-values lower than 0.05 were written bold.

Comparisons of groups with different teeth missing positions and the control group (all normal individuals) are shown in Table 4. Compared with control group, posterior teeth missing group showed more significant results than anterior teeth missing group(*P*=0.021,in genotype for anterior teeth missing group; *P*=0.008, in genotype for posterior teeth missing group; *P*=0.006,in allele for anterior teeth missing group; *P*=0.001, in allele for posterior teeth missing group) and maxillary teeth missing group showed more significant results than mandibular teeth missing group(*P*=0.003,in genotype for maxillary teeth missing group; *P*=0.035, in genotype for mandibular teeth missing group; *P*=0.001,in allele for maxillary teeth missing group; *P*=0.008, in allele for mandibular teeth missing group). Additionally, compared with control group, premolar teeth missing group showed more significant results than incisor teeth missing group, canine teeth missing group and molar teeth missing group (*P*=0.005, in genotype for premolar teeth missing group; *P*=0.001, in allele for premolar teeth missing group). 

**Table 4:** Genotype and allele frequencies of the rs929387 in the control group and missing teeth groups.

Samples	Genotype				Allele			
	TT	TC	CC	*P*-value	T	C	*P*-value	OR(95%CI)
Control	127(72.6%)	43(24.5%)	5(2.9%)		297(84.9%)	53(15.1%)		
Anterior teeth missing	161(59.9%)	94(34.9%)	14(5.2%)	**0.021**	416(77.3%)	122(22.7%)	**0.006**	1.643(1.152-2.344)
Posterior teeth missing	100(57.8%)	60(34.7%)	13(7.5%)	**0.008**	260(75.1%)	86(24.9%)	**0.001**	1.854(1.267-2.712)
Maxillary teeth missing	97(55.4%)	67(28.3%)	11(6.3%)	**0.003**	261(74.6%)	89(25.4%)	**0.001**	1.911(1.309-2.790)
Mandibular teeth missing	182(51.5%)	96(32.4%)	18(6.1%)	**0.035**	460(77.7%)	132(22.3%)	**0.008**	1.608(1.133-2.283)
Incisor teeth missing	153(61.5%)	88(35.3%)	8(3.2%)	0.054	394(79.1%)	104(20.9%)	**0.034**	1.479(1.028-2.127)
Canine teeth missing	51(59.3%)	32(37.2%)	3(3.5%)	0.092	134(77.9%)	38(22.1%)	**0.049**	1.589(0.999-2.527)
Premolar teeth missing	94(56.6%)	59(35.6%)	13(7.8%)	**0.005**	247(74.4%)	85(25.6%)	**0.001**	1.928(1.316-2.826)
Molar teeth missing	55(56.7%)	37(38.1%)	5(5.2%)	**0.028**	147(75.8%)	47(24.2%)	**0.009**	1.792(1.154-2.781)

OR: Odd Ratio; CI: confidence intervals; P-values lower than 0.05 were written bold.

### Point mutation caused by rs929387 lead to a reduced transcriptional activity of GLI3

**Figure 1:**
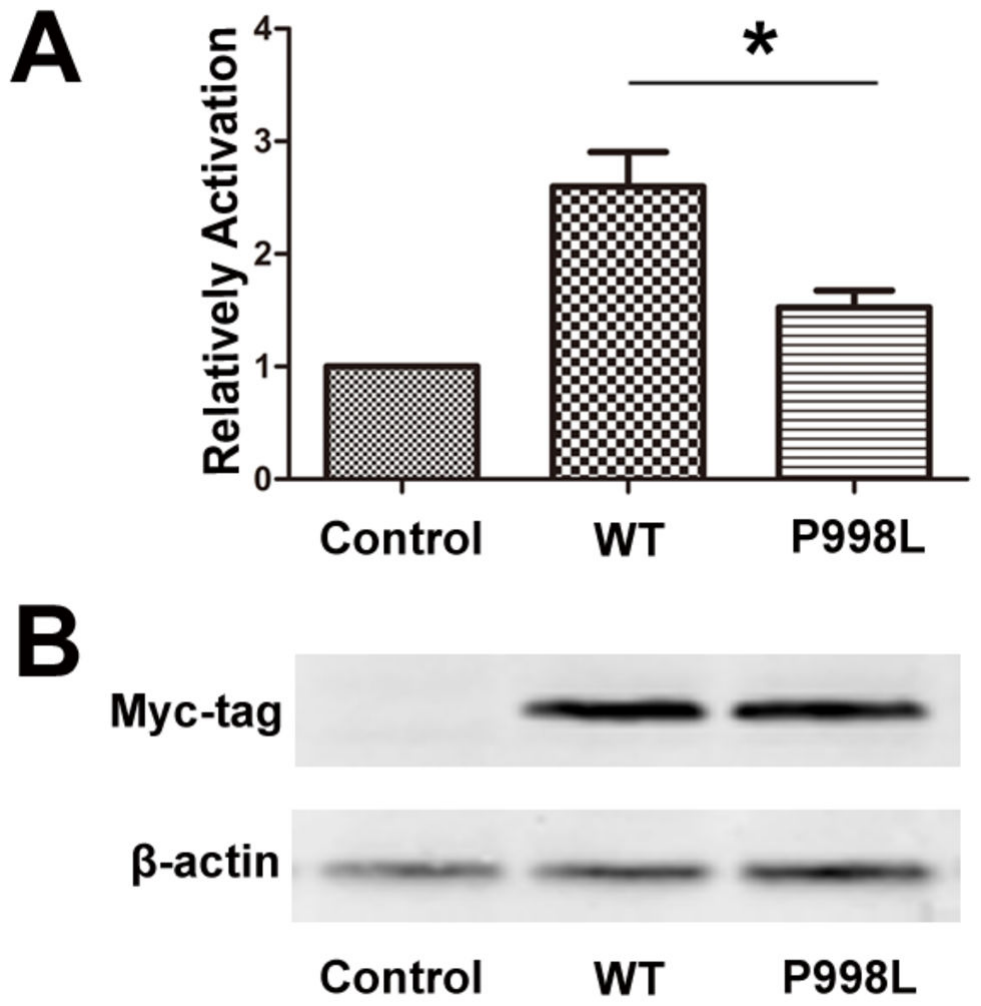
GLI3-reporter assay. (a) Firefly-luciferase under the control of GLI-binding site (GLI-BS) was co-transfected either with wild-type (WT) or mutant (P998L) myc-GLI3. As an internal transfection control renilla-luciferase was included and used for normalization. Values are mean±SD of three independent experiments performed in triplicate. Error bars represent the standard deviations of the mean; *P <0.05. (b) Western blot of lysates from (a). Similar expression levels of wt and mutant Myc-GLI3 are shown by detection with anti-Myc antibodies (upper panel). Loading of equal protein amounts is shown by β-actin-staining (lower panel).

To evaluate the transactivation activity of the mutant GLI3 proteins caused by rs929387, we performed luciferase reporter assay experiments in HeLa cells, using the GLI-BS Luc plasmid as reporter. GLI-BS Luc plasmid contained 8 directly repeated copies of a GLI-binding site [21]. The mutant GLI3 protein had decreased capabilities to induce the luciferase signal, suggesting that mutant GLI3 caused by rs929387 leads to a decrease in its transcriptional activity (Figure 1a). Equal transfection and synthesis efficiencies of the wt and mutant GLI3-constructs were controlled on a Western blot analyzing ratios of expressed GLI3 to β-actin (Figure 1b). This result suggested that rs929387 polymorphism may affect the function of GLI3 protein.

## Discussion

Many factors, including environmental and genetic factors, multi-reagent chemotherapy and radiotherapy, may contribute to tooth agenesis [22]. Although the exact mechanism of tooth agenesis has not been fully elucidated, genetic factors are believed to play a major role in tooth agenesis. The incidence of tooth agenesis is very high (ranges from 2.2 to 10.1%) [2], however, only a few cases can be linked to gene mutation [1]. This suggests that tooth agenesis may be a polygenic disease. Hundreds of genes have been associated with tooth development and can potentially contribute to tooth agenesis. These genes code for signaling molecules, transcription factors, and factors controlling cell proliferation and differentiation [23]. Individuals with distinct polymorphic alleles may exhibit subtle and specific phenotypic variations in dental patterning. Consequently, it can be speculated that association studies between gene polymorphisms and hypodontia as well as other mild malformations will reflect qualitative defects of embryogenesis [24]. Therefore, we focus on the association between tooth agenesis and single nucleotide polymorphisms. 

 In a previous study, we found the association between twors929387 of *GLI3* and non-syndromic hypodontia [20]. In this study, we investigated the potential function of rs929387 in oligodontia individuals (absence of more than six teeth). The results further confirm our previous conclusion that polymorphisms on rs929387 of *GLI3* may be a risk factor for Chinese Han population with tooth agenesis. Oligodontia group was also investigated in this study. We found that the difference for genotype and allele frequencies was more significant in oligodontia group than in hypodontia group. The data demonstrate a strong relationship between the marker rs929387 of *GLI3* and sporadic oligodontia tooth agenesis in the Han population and implicated allele C as its risk factor.

Interestingly, following stratification of the case and control groups on the basis of gender, comparisons revealed marked differences in rs929387 between the gender groups than between all case-control groups. We found that the difference was more significant in males than in females. Actually, no significant difference was observed in female groups. It suggests that rs929387 may be a risk factor for male Han population. 

The position of missing teeth may also contribute to this difference. The frequency of allele C was higher in posterior teeth missing group and maxillary teeth missing group. Also compared results among different type of teeth missing group (incisor, canine, premolar and molar), frequency of allele C was highest in premolar teeth missing group than others. Our results shows rs929387 of *GLI3* may have more close relationship with posterior and maxillary teeth missing. Tooth development is known to be a complex process in which different genes are involved in the development of each tooth [23]. The results of this study are in accordance with this point. 

The GLI3 protein is a zinc finger transcription factor expressed in early development. This transcription factor regulates downstream genes by direct binding to specific sequences in the promoter region of target genes [25]. The GLI3 protein is a downstream mediator of the sonic hedgehog pathway, and this pathway includes several genes that cause abnormal phenotypes in the human when mutated (for example, *SHH*, *PTC1*, and *CBP*) [26]. Shh pathway is involved in both lateral (epithelial-mesenchymal) and planar (epithelial-epithelial) signaling in early tooth development and GLI3 is expressed in both the epithelial and mesenchymal layers. A recent study demonstrates the expression of SHH signaling in the developing human tooth and suggests a conserved function of SHH signaling pathway during human odontogenesis [27]. Thus, we assume that variation in *GLI3* may affect teeth development via SHH signaling pathway. The marker rs929387 (c.2993C→T) is located in exon14 of GLI3 and includes a C→T transversion resulting in Pro 998 Leu. Rs929387 is located in the transactivation and CBP-binding regions of GLI3 [28]. By luciferase reporter assay test, we found variation in GLI3 caused by rs929387 could reduce transcriptional activity of GLI3. This test further confirmed that polymorphism in rs929387 may affect the function of GLI3 and then affect the development of teeth. However, the specific mechanism is not clear and more experiment need to be done to reveal the mechanism of this complex process. 

Previous studies on polymorphisms and tooth agenesis was very rare. One of our studies suggests the potential relationship between polymorphism in rs929387 of GLI3 and non-syndromic hypodontia. Another recent report found significant difference between rs929387 and hypodontia in a Turkey family-based analysis [29]. However, in the Brazilian case-control study, they didn’t find the same relationship. This may provide a clue that polymorphism in rs929387 may associate with tooth agenesis not only in Chinese Han population but also in other ethnic groups and their relationship was different among groups.

The human dentition develops in a long process that starts during the second month of embryogenesis and is completed during adolescence when the third molars (‘‘wisdom teeth’’) erupt. The process is regulated by tissue interactions and genetic networks. Genetic factors (mutation or polymorphism) can explain some causes of tooth agenesis. Also there Epigenetics, an area of research that is studying how environmental factors produce lasting changes in gene expression without altering DNA sequence, may provide new insights into this question.

In summary, in this study, we demonstrate that polymorphism in rs929387 of *GLI3* may contribute to the sporadic non-syndromic tooth agenesis in Chinese Han people. Our gene functional study shows that polymorphism in rs929387 affected the expression of *GLI3* gene. However, more experiments may be need to elucidate the regulatory mechanism of GLI3-mediated tooth agenesis.
